# Association of pre-eclampsia risk with maternal levels of folate, homocysteine and vitamin B12 in Colombia: A case-control study

**DOI:** 10.1371/journal.pone.0208137

**Published:** 2018-12-06

**Authors:** Norma C. Serrano, Doris Cristina Quintero-Lesmes, Silvia Becerra-Bayona, Elizabeth Guio, Mónica Beltran, María C. Paez, Ricardo Ortiz, Wilmar Saldarriaga, Luis A. Diaz, Álvaro Monterrosa, Jezid Miranda, Clara M. Mesa, José E. Sanin, German Monsalve, Frank Dudbridge, Aroon D. Hingorani, Juan P. Casas

**Affiliations:** 1 Fundación Cardiovascular de Colombia, Floridablanca, Colombia; 2 Hospital Internacional de Colombia, Piedecuesta, Colombia; 3 Universidad Autónoma de Bucaramanga, Bucaramanga, Colombia; 4 Universidad Industrial de Santander, Bucaramanga, Colombia; 5 Departamento de Ginecología y Obstetricia, Departamento de Morfología, Facultad de Salud, Universidad del Valle, Cali, Colombia; 6 Universidad de Cartagena, Cartagena, Colombia; 7 Universidad CES, Medellín, Colombia; 8 Universidad Pontificia Bolivariana, Bucaramanga, Colombia; 9 Department of Health Sciences, Centre for Medicine, University of Leicester, Leicester, United Kingdom; 10 Farr Institute of Health Informatics, University College London, London, United Kingdom; University of Missouri Columbia, UNITED STATES

## Abstract

**Background:**

Maternal serum concentrations of folate, homocysteine, and vitamin B12 have been associated with pre-eclampsia. Nevertheless, reported studies involve limited number of cases to reliably assess the nature of these associations. Our aim was to examine the relation of these three biomarkers with pre-eclampsia risk in a large Colombian population.

**Materials and methods:**

***Design***: A case-control study.

***Setting*:** Cases of pre-eclampsia and healthy pregnant controls were recruited at the time of delivery from eight different Colombian cities between 2000 and 2012.

***Population or Sample***: 2978 cases and 4096 controls were studied. Maternal serum concentrations of folate, homocysteine, and vitamin B12 were determined in 1148 (43.6%) cases and 1300 (31.7%) controls. Also, self-reported folic acid supplementation was recorded for 2563 (84%) cases and 3155 (84%) controls.

***Analysis*:** Adjusted odds ratios (OR) for pre-eclampsia were estimated for one standard deviation (1SD) increase in log-transformed biomarkers. Furthermore, we conducted analyses to compare women that reported taking folic acid supplementation for different periods during pregnancy.

***Main Outcomes Measures***: Odds ratio for pre-eclampsia.

**Results:**

After adjusting for potential confounders in logistic regression models, the OR for pre-eclampsia was 0.80 (95% CI: 0.72, 0.90) for 1SD increase in log-folate, 1.16 (95%CI: 1.05, 1.27) for 1SD increase in log-homocysteine, and 1.10 (95%CI: 0.99, 1.22) for 1SD increase in log-vitamin B12. No interactions among the biomarkers were identified. Women who self-reported consumption of folic acid (1 mg/day) throughout their pregnancy had an adjusted OR for pre-eclampsia of 0.86 (95%CI: 0.67, 1.09) compared to women that reported no consumption of folic acid at any point during pregnancy.

**Conclusions:**

Maternal serum concentrations of folate were associated as a protective factor for pre-eclampsia while concentrations of homocysteine were associated as a risk factor. No association between maternal vitamin B12 concentrations and preeclampsia was found.

## Introduction

Pre-eclampsia is defined as the development of hypertension and proteinuria after the 20th week of gestation in normotensive women [1], and is considered one of the major causes of maternal and neonatal morbidity and mortality in low and middle-income countries [2–3]. Although the aetiology of this disorder is poorly understood, a great amount of effort has been made to identify potential causal risk factors toward preventing the development of pre-eclampsia [4–7]; nevertheless, they have not yet been translated into largely effective interventions [8–10].

In the last two decades, a number of authors have investigated the effect that maternal concentrations of folate, homocysteine, and vitamin B12 may have on the development of pre-eclampsia [11–12]. Accumulating evidence suggests that hyperhomocysteinemia may be a cause of the endothelial dysfunction provoked by oxidative stress in pre-eclampsia [13–14]. Homocysteine is an intermediate amino acid that is metabolized into cysteine or methionine. In the latter, homocysteine requires folate as a co-substrate and vitamin B12 as a cofactor for the function of involved enzymes such as the methionine synthase enzyme [12]. Low levels of folate and vitamin B12 have been linked to higher homocysteine levels in the bloodstream, increasing the risk of pre-eclampsia [14].

Experimental *in vitro* studies have suggested that high levels of homocysteine induce apoptosis and up-regulation of antiangiogenic factors in human trophoblastic cells obtained from placentas of healthy pregnant women [15–17]. Interestingly, some of these processes appear to be suppressed or reverted when trophoblastic cells are treated with folic acid [18–19] In particular, *in vitro* studies have shown that human trophoblastic cells obtained from placental tissue during early pregnancy, promote an increase in extravillous trophoblast invasion and vascular density (angiogenesis) when cultured in presence of folic acid [19].

Despite these *in vitro* approaches provide some evidence to the hypothesis that low levels of folate may lead to pre-eclampsia, observational studies elucidating its impact are deficient [12]. This situation is mainly due to the limited sample size they include as well as the inadequate control that is performed for potential confounding since most authors only report unadjusted differences in levels of biomarkers by case control status [12].

Likewise, further limitations include a lack of power to not only evaluate the association of biomarkers with pre-eclampsia sub-phenotypes but also determine the shape of this association among them and explore possible interactions between folate and homocysteine. In an effort to overcome these limitations, we investigated the relationship of maternal levels of three biomarkers (folate, homocysteine, and vitamin B12) with pre-eclampsia using a large case-control study in pregnant women from Colombia. The understanding gained from this research will contribute to propose randomised trials that include folic acid supplements to prevent the development of this disorder.

## Materials and methods design

The population included in this work is part of the case-control Genetics and Pre-eclampsia (GenPE) study conducted in eight different Colombian cities [20]. Participants were young primigravid women recruited at time of delivery between December 2000 and February 2012. Pre-eclampsia was defined as blood pressure ≥ 140/90mmHg and proteinuria ≥ 300mg/L in 24-hour specimens or ≥ 2+ dipstick reading in random urine samples, evaluated after 20 weeks of gestation without evidence of any urinary tract infection. Controls were healthy normotensive women without proteinuria, recruited at term (gestation had lasted ≥ 37 weeks). Also, a control was assigned to a case by assuring that both were from the same recruitment centre, and if possible, had the same ethnicity and gestational age (±1 week).

GenPE study recruited 3702 cases and 4705 controls. All cases and controls were validated by an outcome committee before their final inclusion in the study. For this project, women older than 25 years old (n = 232), women without information on biomarker levels or folic acid supplementation (n = 675), and women without data on maternal age, gestational age, or ethnicity (n = 104) were excluded, leaving available information from 2978 cases and 4096 controls for analysis.

Women with a history of autoimmune, metabolic (including diabetes or gestational diabetes), renal, or cardiovascular (including chronic hypertension) disease were excluded of the study. For subsidiary analyses, pre-eclampsia cases were divided into severe or mild pre-eclampsia and early- or late-onset pre-eclampsia. Severe pre-eclampsia was defined as blood pressure >160/110 mmHg and proteinuria >5 g/day or 3+ dipstick, while mild pre-eclampsia was considered when neither blood pressure nor proteinuria reached these thresholds. Likewise, early and late pre-eclampsia were defined as that occurring before or after 34 weeks of gestation, respectively.

### Data collection

**Fig 1:**
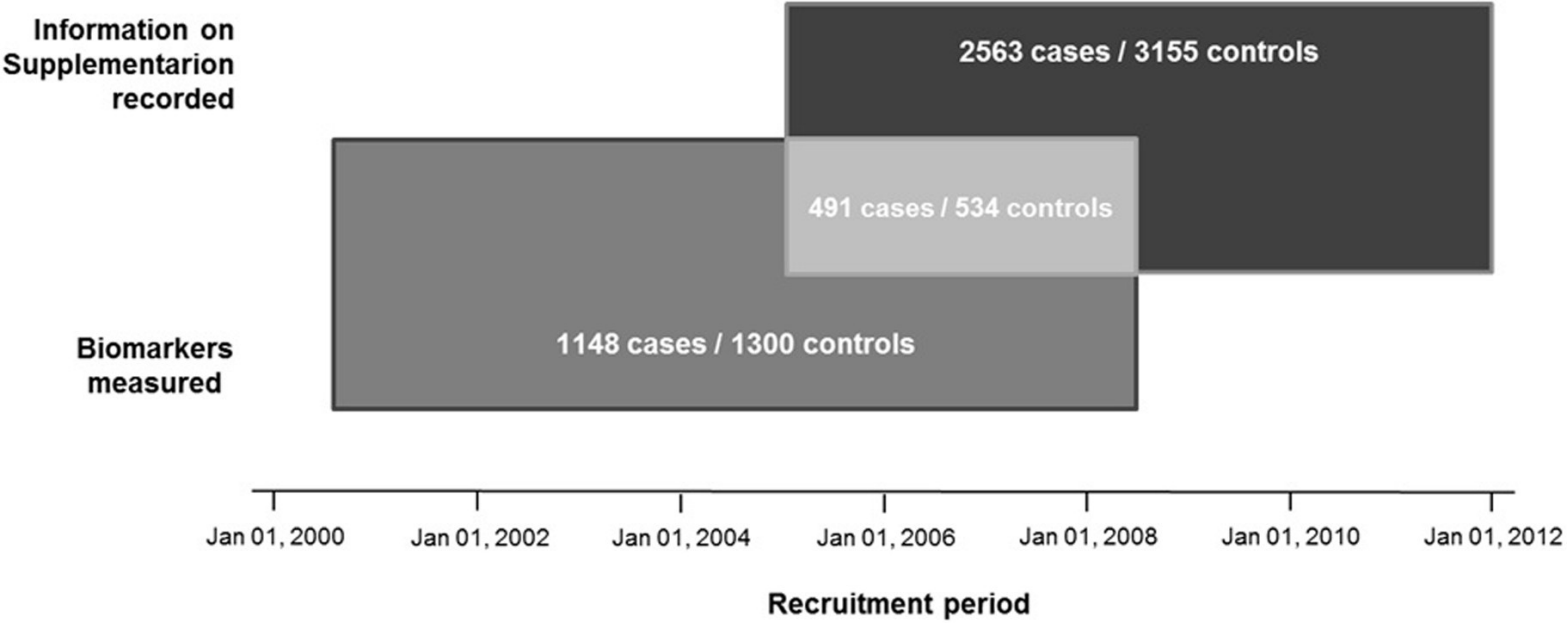
Recruitment period and availability of data for participants in the GenPE study. Each rectangle depicts the total number of cases and controls with available information for the studied biomarkers and folic acid supplementation. Participants with available information for both are shown in the overlapping region of the rectangles.

Trained personnel conducted a verbal interview to collect clinical and demographic data from participants through an in-depth questionnaire. Questions related to folic acid supplementation during pregnancy were included after July 2005 (Fig 1).

Similarly, folate, homocysteine, and vitamin B12 levels were only measured in samples from participants recruited between December 2000 and November 2008 (Fig 1). Maternal serum concentrations of folate, homocysteine, and vitamin B12 were determined in 1148 (43.6%) cases and 1300 (31.7%) controls.

To measure biomarker levels, blood samples were taken at the time of recruitment from the antecubital vein, and their components were separated within 1 hour. Serum was extracted, frozen, and maintained at –80°C until performing the assays. Levels of folate, homocysteine, and vitamin B12 were measured by competitive protein binding chemiluminescent assays following manufacturer’s instructions (IMMULITE; Siemens Medical Solutions Diagnostics). The assay detection ranges were 2–50 μmol/L for homocysteine, 1–24 ng/mL for folate, and 150–1000 pg/mL for vitamin B12.

In addition, assays were conducted by laboratory technicians blinded to participants’ disease outcome, and case and control samples were randomly distributed across plates to minimize potential assay bias. For quality control purposes, measurements were repeated within 24 hours of the first quantification in randomly selected blood samples as follows: 26 cases and 38 controls in the case of homocysteine, 29 cases and 28 controls in the case of folate, and 21 cases and 28 controls for vitamin B12.

Ethics approval was obtained prior to participant recruitment by the institutional Ethics Review Board of Universidad Autónoma de Bucaramanga, Colombia (Research Ethics Committee. Act No. 0037/2007. August 29, 2007). All participants signed a written, inform consent. Participants under age 18 also signed this consent as well as their legal guardian.

### Statistical analysis

Data were described using means and standard deviations (SD) for continuous variables and counts (percentages) for categorical variables. Differences between cases and controls were evaluated using the t-test and the Kruskal-Wallis test for normally and non-normally distributed continuous variables, respectively. For discrete variables, the Chi-squared test or the Fisher exact test were used to compare proportions. This comparison across multiple groups (i.e. controls, early and late onset and severe PE, was conducted using ANOVA.

Multiple imputations were performed for the observations with incomplete information related to maternal serum concentrations of folate, homocysteine and vitamin B12 in order to minimize variations in the estimations. Hence, sample proportions with values outside the detection limits of the assay were 4% for folate, 3% for homocysteine, and 39% for vitamin B12 (S1 Table). Due to the significant sample proportion obtained below the detection limit for vitamin B12, multiple imputations were used for these values in order to minimize variations in the estimations (S1 Appendix), instead of excluding them or performing simple substitutions. The latter has shown to produce altered results when the proportion of values outside the detection limit is greater than 30% [21–22]. For the quality control samples, Lin's concordance correlation coefficient [23] was estimated for both measurements, and indicated a complete agreement between them; specifically, 1.0 (95%CI: 0.99, 1.0) for folate, 0.98 (95%CI: 0.97, 0.99) for homocysteine, and 1.0 (95%CI: 1.0, 1.0) for vitamin B12.

Multiple logistic regression was used to evaluate the association between maternal serum concentrations of folate, homocysteine and vitamin B12, and pre-eclampsia. Crude odds ratios (OR), 95% confidence intervals (95%CI) and in some cases, standard deviation, were estimated for each blood marker. Likewise, estimates were adjusted for maternal age, ethnicity, socioeconomic status, multiple pregnancy, smoking, infections during pregnancy, recruitment centre, and recruitment year. In order to evaluate possible changes of the effect of the biomarkers over time, interactions between the biomarkers and the year of recruitment (*i*.*e*. year of sampling) were tested in multivariable models the likelihood ratio test (LRT) (comparing nested models with and without the interaction term). For sub-phenotype analyses, all biomarkers were log-transformed, standardized, and introduced as continuous variables into adjusted logistic models that simultaneously estimated the association of each metabolite with the presence of early- and late-onset pre-eclampsia.

#### Dose response association

The association of maternal serum concentrations of folate, homocysteine, and vitamin B12 with pre-eclampsia was assessed in logistic regression models by introducing the biomarkers as continuous variables after logarithmic transformation (given their positively-skewed distributions). Restricted cubic spline functions were used to evaluate the dose response in these models after adjusting for maternal age, gestational age, recruitment centre, and ethnicity. The spline functions were fitted with three internal knots since this provided the best fit (S1 Appendix). Departures from linearity were determined through LRT [24] by comparing models with and without the nonlinear components of the splines.

#### Association analyses of biomarker levels with pre-eclampsia

Logistic regression models followed progressive adjustment accounting for different maternal variables such as age, recruitment centre, recruitment date year-over-year, self-reported ethnicity, multiple pregnancy, smoking, socioeconomic position (in two categories), infections during pregnancy, and gestational age (in weeks). The association of maternal folate levels and pre-eclampsia was also adjusted by folic acid supplementation in those participants that had information for both (Fig 1). Odds ratios and 95% confidence intervals were reported.

Two potential effect modifications were also examined. The first evaluated the influence that folate and vitamin B12 levels may have on the association between homocysteine and pre-eclampsia (interaction previously reported for other diseases) [25]; and the second investigated the effect that the recruitment period (included as a year-over-year change) may have on the association of each biomarker with pre-eclampsia. The second method may give some insight of the consequences related to the introduction of folic acid fortification policies or changes in pregnancy care over time within the recruitment period. Interaction terms (in a multiplicative scale) were introduced into the fully adjusted logistic regression models, which were compared against models without the interaction terms using the LRT (24). A p value lower than 0.05 (p < 0.05) indicated that the nested model with the interaction term had a better fit.

#### Folic acid supplementation and pre-eclampsia

For participants recruited between July 2005 and February 2012, the association of self-reported folic acid supplementation (1 mg/day) on pre-eclampsia risk was evaluated using logistic regression models. Dummy indicators were generated according to the period of supplementation in trimesters [none (reference), one to two, or three] (S2 Table). The effect of preconception supplementation was not assessed due to only 0.5% of women reported its consumption. Possible effect modification by recruitment date (included as a year-over-year change) was evaluated with interaction terms tested by the LRT. The validity of self-reported folic acid supplementation was assessed through looking at differences in biomarker levels by categories of the period of supplementation. Adjusting the effect of folic acid supplementation by maternal folate was considered unnecessary since it lies in the causal pathway from exposure to outcome, and the design of the study did not allow evaluating such mediation pathways [26] (S1 Fig).

#### Sub-analysis by pre-eclampsia early vs. late, mild and severe

As a subsidiary analysis, the magnitude of the associations for biomarker levels and folic acid supplementation was evaluated among pre-eclampsia sub-phenotypes in multinomial logistic regression models. In these analyses, subgroups of pre-eclampsia defined by severity (mild and severe cases) and time of onset (late and early pre-eclampsia) were compared simultaneously to the control group. Multinomial logistic regression, an extension of binary logistic regression, was used to estimate the effect of the same risk factor in two pre-eclampsia categories within a single model by allowing to include more than two categories of the outcome variable (mutually exclusive) [27]. Since gestational age defines the presence of early or late pre-eclampsia, it was removed from the models that evaluate the biomarkers and any pre-eclampsia subgroup in order to permit the comparison between estimates.

All estimates and 95% confidence intervals (95% CI) were calculated incorporating the complex design of the sample into the Stata software, version 14 (Stata Corporation, College Station, TX).

## Results

### Descriptive analysis

For this study, complete data for 2978 cases and 4096 were included. Sociodemographic and clinical variables of participants are shown in Table 1. Data revealed that over 90% of the participants belonged to a low socioeconomic status, the percentage of multiple pregnancies was extremely low (1.0% of the total), and there was a very small number of smokers (2.0% of the total).

**Table 1:** Baseline characteristics of participants from the GenPE study.

Variable	Cases (n = 2978)	Controls (n = 4096)	*p-value*
Maternal age (years), mean (SD)	20.0	18.6 (2.8)	<0.001†
Gestational age (weeks), mean (SD)	36.8 (3.3)	39.1 (1.2)	<0.001†
Systolic blood pressure (mmHg), mean (SD)	151.5 (13.4)	111.0 (8.7)	<0.001†
Diastolic blood pressure (mmHg), mean (SD)	100.1 (10.0)	68.6 (7.4)	<0.001†
Folic Acid (ng/mL), mean (SD)	6.4 (4.3)	6.6 (4.1)	<0.001†
Homocysteine (μmol/L), mean (SD)	8.4 (4.9)	8.0 (4.8)	<0.001†
Vitamin B12 (pg/mL), mean (SD)	287.2 (158.7)	250.4 (127.1)	<0.001†
Multiple pregnancies, n (%)			
Yes	60 (2.0)	8 (0.2)	<0.001†
No	2917 (98.0)	4086 (99.7)	
Missing	1 (0.03)	2 (0.1)	
Ethnicity, n (%)			
White Hispanic	417 (14.2)	538 (13.1)	<0.001†
Afro-Caribbean	502 (16.6)	501 (12.2)	
Amerindians	45 (1.6)	58 (1.4)	
Mixed	2014 (67.7)	2999 (73.2)	
Missing	0 (0.0)	0 (0.0)	
Socioeconomic status, n (%)			
Low (<3)	2658 (88.9)	3646 (89.0)	0.838†
Medium-High (>3)	275 (9.6)	373 (9.1)	
Missing	45 (1.6)	77 (1.9)	
Smoking during pregnancy, n (%)			
Yes	52 (1.8)	84 (2.1)	0.280†
No	2909 (97.7)	3995 (97.5)	
Missing	17 (0.6)	17 (0.4)	
Infections during pregnancy, n (%)			
Yes	2085 (69.6)	2738 (66.9)	0.002†
No	822 (28.1)	1302 (31.8)	
Missing	71 (2.3)	56 (1.4)	
Folic acid supplementation (at any time), n (%)			
Yes	2563 (86.1)	3155 (77.0)	0.829†
No	412 (13.8)	486 (11.8)	
Not collected*	3 (0.1)	455 (11.2)	

* Data was not collected because a different version of the recruitment questionnaire was used. In this version, the corresponding questions were not included.

† p-values correspond to one-way ANOVA test for continuous variables and Chi-squared test for categorical variables (excluding missing p-values).

SD: standard deviation.

From the 2978 cases, 731 were classified as severe pre-eclampsia and 2247 as mild pre-eclampsia. Similarly, according to time of presentation, 406 cases were early pre-eclampsia, and 2572 were late pre-eclampsia.

Also, there were 1148 cases and 1300 controls with available data on maternal serum concentration for at least one biomarker (1077 cases and 1276 controls with data for all three), and 491 cases and 534 controls with values for at least one biomarker plus information on folic acid supplementation during pregnancy (Fig 1).

### Biomarker levels

#### Dose response association

For the three biomarkers, the shape of the association was consistent with a linear relationship between log-transformed values and log-odds of pre-eclampsia (with the spline models showing no departures from linearity: p = 0.327 for log-folate, p = 0.450 for log-homocysteine, and p = 0.309 for log-vitamin B12). Log-folate was inversely associated with pre-eclampsia risk, while a positive linear association was observed for log-homocysteine and log-vitamin B12. Given that a linear term for log-values was sufficient to summarize the associations under investigation, ORs and 95%CI were reported for 1 standard deviation (SD) change in log-values (Table 2).

**Table 2:** Association dose response of log-folate, log-homocysteine and log-vitamin B12 with pre-eclampsia (adjusted).

Adjustment	n	Folate OR (95%CI)	p-value	n	Homocysteine OR (95%CI)	p-value	n	Vitamin B12 OR (95%CI)	p-value
Maternal age (years)	2374	0.91 (0.84, 0.99) *	0.021	2380	1.18 (1.09, 1.28) *	<0.001	2374**	1.22 (1.12, 1.33) *	<0.001
Plus recruitment center (eight categories)	2373	0.89 (0.81, 0.98)	0.021	2379	1.15 (1.04, 1.27) *	0.005	2373**	1.22 (1.11 1.31) *	<0.001
Plus recruitment date (years)	2373	0.82 (0.74, 0.91) *	<0.001	2379	1.14 (1.04, 1.26) *	0.008	2373**	1.20 (1.11, 1.28) *	<0.001
Plus ethnicity (four categories)	2373	0.82 (0.74, 0.92) *	<0.001	2379	1.14 (1.04, 1.26) *	0.008	2373**	1.19 (1.07, 1.26) *	<0.001
Plus low SES (low *vs*. medium-high)	2344	0.82 (0.73, 0.91) *	<0.001	2350	1.15 (1.04, 1.27) *	0.005	2373**	1.20 (1.12, 1.27) *	<0.001
Plus multiparity (yes *vs*. no)	2330	0.82 (0.73, 0.91) *	<0.001	2336	1.15 (1.04, 1.27) *	0.006	2358**	1.19 (1.10, 1.25) *	<0.001
Plus smoking (yes *vs*. no)	2330	0.82 (0.73, 0.91) *	<0.001	2336	1.15 (1.04, 1.27) *	0.006	2358**	1.19 (1.10, 1.24) *	<0.001
Plus infections (yes *vs*. no)	2374	0.85 (0.77, 0.93) *	0.001	2379	1.18 (1.08, 1.29) *	<0.001	2374**	1.18 (1.54, 1.23) *	<0.001
Plus gestational age (weeks)	2270	0.80 (0.72, 0.90) *	<0.001	2276	1.16 (1.05, 1.27) *	0.010	2298**	1.10 (0.99, 1.22) *	0.079
Plus FAS (three categories)	1231	0.88 (0.75, 1.04) *	0.125	1223	1.22 (1.07, 1.40) *	0.003	1209**	1.09 (0.95, 1.26) *	0.204

*OR reported for 1SD increase in log-transformed values.

**Multiple imputed data for vitamin B12 values.

FAS: folic acid supplementation.

SES: Socioeconomic status

#### Association analyses

**Fig 2:**
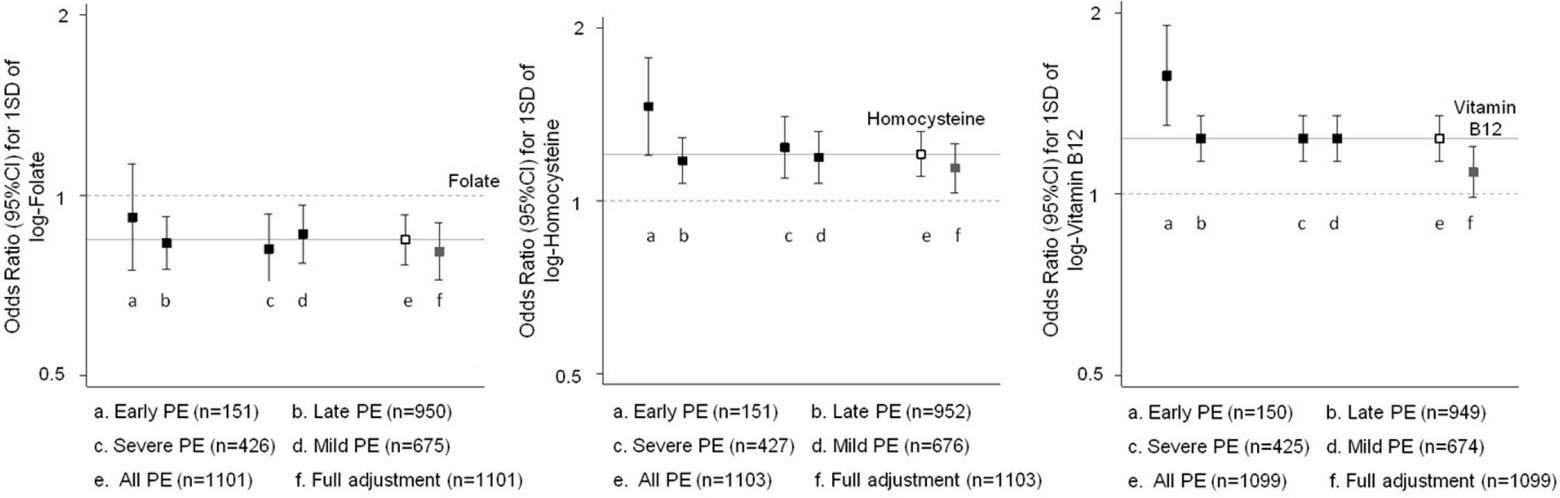
Sensitivity analysis for the effect of folate, homocysteine and vitamin B12 according to pre-eclampsia (PE) severity and time of onset. The following variables were included in the full adjustment: age, recruitment centre, recruitment date year-over-year, self-reported ethnicity, multiple pregnancy, smoking, socioeconomic position (in two categories), infections during pregnancy, and gestational age (in weeks).

All potential confounders included in the logistic models had a very low percentage of missing values (< 2.5%). The fully-adjusted OR for 1SD increase of log-folate was 0.80 (95%CI: 0.72, 0.90). The subsidiary analysis by sub-phenotypes of pre-eclampsia showed no strong evidence of these associations to be different from the one obtained for all pre-eclampsia cases (Fig 2). The sub-sample (56% of total sample size) adjustment for folic acid supplementation showed a similar point estimate; however, the confidence interval was wider (OR of 0.88; 95%CI: 0.75, 1.04).

In the fully-adjusted model, the OR for an increase of 1SD in log-homocysteine was 1.16 (95%CI: 1.05, 1.27). Estimates were consistent among pre-eclampsia sub-phenotypes defined by pre-eclampsia severity and time of onset (Fig 2). No interactions were observed with maternal levels of folate (p = 0.191) or vitamin B12 (p = 0.568).

The association between 1SD increase in log-vitamin B12 and pre-eclampsia risk was considerably attenuated from 1.22 (95%CI: 1.12, 1.33) in the age-adjusted model to 1.10 (95%CI: 0.99, 1.22) in a fully-adjusted model. Similar results were obtained after excluding the multiple imputed data (S3 Table). The estimates from pre-eclampsia sub-phenotypes were largely consistent with those observed for all pre-eclampsia (Fig 2).

On the other hand, there was no evidence of effect modification by year of recruitment for any of the three biomarkers (p = 0.908 for folate; p = 0.252 for homocysteine; p = 0.874 for vitamin B12).

#### Low-dose folic acid supplementation

From the controls with information on folic acid supplementation (1 mg/day), 86% reported consumption at any time during pregnancy, with 16% reporting supplementation continuously throughout the entire pregnancy and 70% reporting shorter periods of supplementation. In a sub-sample of 534 controls, we compared those women who consumed supplements during all pregnancy versus those who took supplements during short periods of pregnancy. Results revealed that women who consumed folic acid throughout pregnancy had higher maternal serum concentrations of folate [2.3 ng / mL (95% CI: 0.9, 3.6)] compared to those that took folic acid during short periods. Nevertheless, the latter had an increase of 1.0 ng / mL (95% CI: 0.4, 1.6) in maternal serum folic acid concentrations.

**Fig 3:**
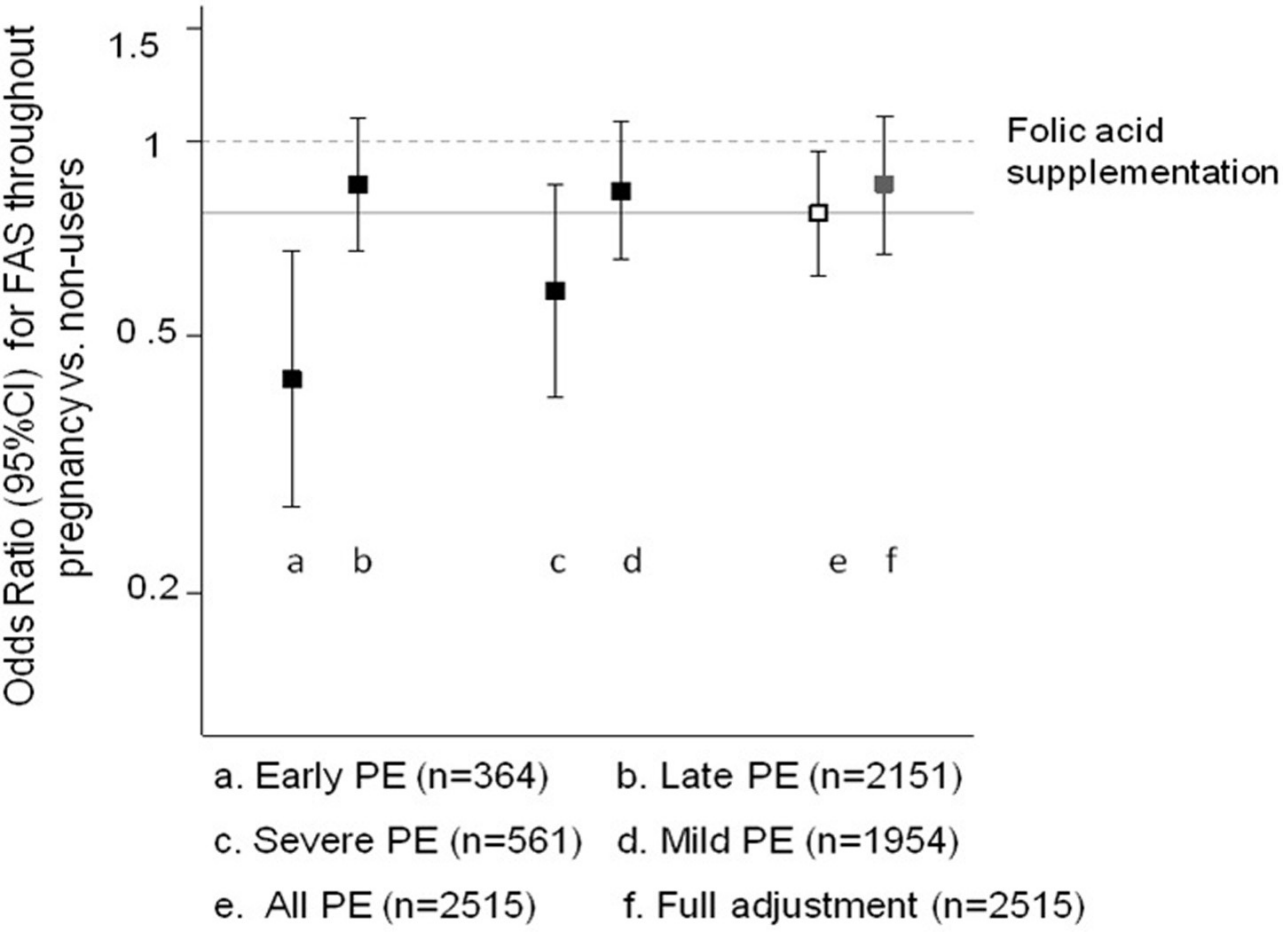
Sensitivity analysis for the effect of folic acid supplementation according to pre-eclampsia (PE) severity and time of onset. The following variables were included in the full adjustment: age, recruitment centre, recruitment date year-over-year, self-reported ethnicity, multiple pregnancy, smoking, socioeconomic position (in two categories), infections during pregnancy, and gestational age (in weeks).

Compared to women who did not reported supplementation, participants who reported use of folic acid supplements for short periods (either one or two trimesters of pregnancy) showed an adjusted OR of 1.00 (95%CI: 0.84, 1.21), and women that reported use of folic acid supplements during the entire pregnancy had an adjusted OR for pre-eclampsia of 0.86 (95%CI: 0.67, 1.09). Subsidiary analysis (comparing supplementation throughout the entire pregnancy vs. never supplemented) showed some evidence of a stronger association with early pre-eclampsia, but for other sub-phenotypes the strength of the association was similar to that reported for all pre-eclampsia (Fig 3). There was no evidence of effect modification by date of recruitment (p = 0.185).

**Fig 4:**
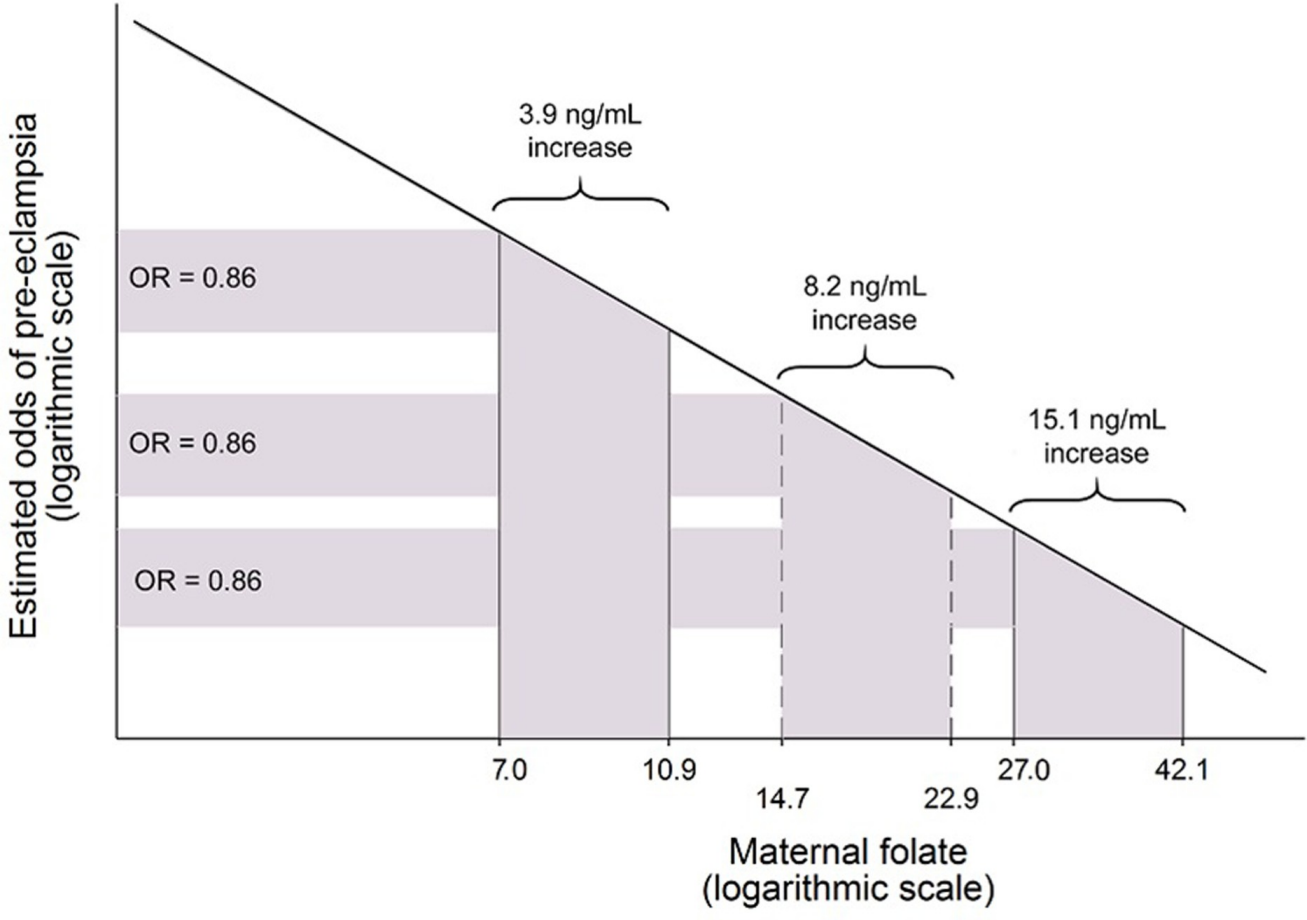
Exposure-response relationship between serum log-folate and pre-eclampsia risk in the GenPE study. This figure presents the absolute increase in folate levels necessary to obtain the same Odds Ratio for pre-eclampsia according to the baseline folate levels. The proportional change in the odds of pre-eclampsia (OR = 0.86) is explained by a proportional increase.

Fig 4 shows the exposure-response relationship between serum log-folate and pre-eclampsia risk in the GenPE study. This figure presents the absolute increase in folate levels necessary to obtain the same Odds Ratio for pre-eclampsia according to the baseline folate levels.

## Discussion

This work examined the association of maternal levels of folate, homocysteine, and vitamin B12 with pre-eclampsia in a large case-control study. Our results showed a possible association for maternal levels of folate and homocysteine with pre-eclampsia, suggesting that higher folate levels lower its risk while higher homocysteine levels increase its risk. On the contrary, the association of vitamin B12 levels with pre-eclampsia was not clear. Subsidiary analysis revealed that the strength of the association was consistent with that observed for all pre-eclampsia cases for most of the pre-eclampsia sub-phenotypes, excluding early pre-eclampsia where the point estimate suggested a stronger association with wider confidence intervals. Also, there was no evidence of interaction between biomarkers.

Despite we showed a strong association of folate and homocysteine levels with pre-eclampsia, the observed results need a careful interpretation since different factors, such as reverse causation, may have an influence on them. Nevertheless, several lines of evidence indicate they are unlikely to entirely explain these observations. To minimize the possibility of confounding, a thorough adjustment for potential confounders was also conducted, suggesting that the associations of folate and homocysteine with preeclampsia are independent of these factors. However, it is well known that observational studies cannot rule out the presence of residual confounding.

Given the fact that folate is ingested from diet and obtained from folic acid supplements, a complementary strategy of analysis was performed to evaluate the influence of folic acid supplementation (1 mg/day) on decreasing the risk of pre-eclampsia in a proportional manner to its effect on folate levels. This analysis revealed that women reporting supplementation of 1 mg/day of folic acid during the entire pregnancy, which increased folate levels by 2 ng/mL, had lower odds of pre-eclampsia by 14% (OR 0.86; 95%CI: 0.67, 1.09). Although with some considerable uncertainty around the point estimate, the observed odds ratio was compatible with the effect on pre-eclampsia predicted by the model derived in the current study [OR of 0.90 (95%CI 0.86, 0.95)] for an increase of 2 ng/mL in folate levels from a baseline of 5.8 ng/mL, that corresponded to the mean folate in women without supplementation. Furthermore, shorter periods of folic acid supplementation was not associated with pre-eclampsia, which is consistent with results reported on the literature for other populations [28–29].

International guidelines endorse the use of low doses of folic acid supplementation at two moments during pregnancy: before and up to the 12th week of gestation to prevent the development of neural tube defects (from 0.4 mg/day up to 1 mg/day), and after this point until the end of the pregnancy to avoid anaemia [30–32]. This latter is recommended for women of low and middle-income countries. In the present study, results strongly suggest a potential benefit for taking *higher* doses of folic acid throughout the *entire pregnancy* to prevent pre-eclampsia. In fact, some authors indicate that the use of folic acid and folic acid-containing supplements offers numerous health advantages for women during pregnancy; for instance, decreasing the risk for gestational hypertension and pre-eclampsia [33–41].

In Colombia, fortification of wheat flour with folic acid (1.54 mg/Kg) was introduced around 1997 [42]; unfortunately, there are no reports on the effectiveness of this intervention at a population level. Furthermore, national guidelines from 2000 recommend folic acid supplementation (1 mg/day) from the time women consider becoming pregnant until the end of the first trimester to prevent the appearance of neural tube defects on children [42]. The same daily dose is also advised to be maintained during the rest of the pregnancy until six months postpartum, along with 60 mg of ferrous sulphate, in order to reduce the prevalence of anaemia. It is important to consider the implications for prevention of the linear response, in log-scale, for maternal folate levels and the odds of pre-eclampsia described in our study. This type of association implies that higher increments in folate levels are needed in folate-replete populations in order to obtain the same relative risk reduction, compared to populations with low average levels.

Extrapolating the results of our log-linear model to other countries, in order to observe a 14% reduction in odds of pre-eclampsia, a trial based in US, where population blood folate average is 10.5 ng/mL [43], would require an increase in folate levels of 5.8 ng/mL in the intervention arm. Likewise, the needed folate increase for the same risk reduction would be 10.7 ng/mL in Canada, where pregnant women with standard care have average folate levels of 19.2 ng/mL [44]. In both cases, the values are two to four-fold higher than those needed in a low and middle income country with average folate levels around 5 ng/mL (Fig 4). In addition, it has been shown that proportional increments in folate levels following folic acid supplementation are greater when pre-treatment concentrations are lower [45]; this means that, for a same dose comparison, both the increase in folate levels and the expected reduction in pre-eclampsia risk are more likely to be smaller in a population with high folate levels.

Several limitations of the present study merit comment. The biomarkers of interest were only available for a random subsample of the study since funding for laboratory assays was insufficient. Similarly, questions related to folic acid supplementation were included in the questionnaire after the GenPe study had started. As a result, the number of participants with both biomarker levels and folic acid supplementation information was limited but sufficient to conduct the analyses.

Regarding the study type (a case-control study), we are unable to examine temporality between the biomarkers and pre-eclampsia, a constraint that is innate to these types of studies. Furthermore, despite detailed self-reported information on risk factors and confounders was available, this information was not validated by linking to medical records. Similarly, the observed associations for folate and homocysteine could be affected by confounding and/or reverse causation; however, several lines of evidence indicate that the latter components are unlikely to entirely explain the findings. To minimize the possibility of confounding, thorough adjustment for potential confounders was conducted, suggesting that associations of folate and homocysteine with pre-eclampsia are independent of these factors; nevertheless, further studies are needed to explore the causality of the observed associations as well as rule out the presence of residual confounding.

## Conclusion

The results of our study suggest that levels of folate are associated with a protective effect for pre-eclampsia and levels of homocysteine are associated with a risk factor for pre-eclampsia. On the contrary, the relationship between levels of vitamin B12 and pre-eclampsia was not clear. Based on these results, randomised trials in low- and middle-income countries are needed to evaluate the efficacy and safety of using high doses of folic acid supplementation during gestational time for pre-eclampsia prevention.

## Supporting information

S1 Appendix Analysis methodology of GenPE data. (DOCX)

S1 Table Values outside the assay detection limit (DL) for homocysteine, folate and vitamin-B12 maternal levels. (DOCX)

S2 Table Folic acid supplementation of participants from the GenPE study during pregnancy. (DOCX)

S3 Table Comparison between models for the association of maternal vitamin B12 with pre-eclampsia using imputed and not-imputed data. (DOCX)

S1 Fig Path diagram association (Directed acyclic graph—DAG) between folic acid supplementation and maternal folate levels with pre-eclampsia. (DOCX)
