# Supplementary material for: Association of pre-eclampsia risk with maternal levels of folate, homocysteine and vitamin B12 in Colombia: A case-control study
Source: PLoS One. 2018 Dec 6;13(12):e0208137. doi: 10.1371/journal.pone.0208137 (PMC6283543; doi:10.1371/journal.pone.0208137)
Supplement: S1 Appendix — (DOCX) [file pone.0208137.s001.docx]

**SUPPORTING INFORMATION**

**S1 Appendix. Analysis methodology of GenPE data**

***A. Imputation Procedures***

Homocysteine and folic acid levels

Values outside the assay detection range were substituted by the value given as the detection limit. This technique was considered appropriate since the proportion of non-detects was less than 5% for both biomarkers.

Vitamin B12

Imputation model by fully conditional specification was fitted including homocysteine, folic acid, and vitamin B12 levels, maternal age, gestational age, recruitment centre, recruitment date, ethnicity, and the disease outcome as covariates. Attenuation of the estimated effects are avoided by including the latter. This model allowed for interactions with time and gestational age and nonlinear effects for the studied biomarkers. Before the imputation process, the biomarkers were normalized using a logarithmic transformation.

Imputation was carried out using the ice module in STATA, which allows for interval regression and specification of censored limits in the distribution of the variables to prevent implausible imputed values. Also, the technique makes distributional assumptions of the variable to be imputed (but no direct assumption on the joint distribution of the variables), and works under the missing variable at random assumption. The imputed values were back-transformed to the original scale before fitting the logistic regression models.

Fifty imputed data sets were generated (20 cycles each) and combined using Rubin’s rule. Sensitivity analysis for the imputations was carried out by comparisons with estimates derived from analyses including only complete cases. The information coming from multiple imputation was only used for the assessment of the association between vitamin B12 and pre-eclampsia risk.

***B. Restricted cubic splines***

The shape of the dose response association between the biomarkers and pre-eclampsia was assessed using restricted cubic spline functions. A spline function is a flexible statistical method useful to model nonlinear relationships in which the observed data is left to determine the form of the association between exposure and outcome. The continuous predictor is transformed using cubic polynomials in a piecewise manner, with the different intervals being smoothly jointed at defined knots. The two end segments are restricted to be linear to decrease the influence of extreme values. Increasing the number of knots usually increases the fit to the data, but this may lead to model overfitting. In this study, the number of internal knots was chosen as the number that gave the lowest Akaike and Bayesian information criteria ^[[1]](#footnote-1),^ ^[[2]](#footnote-2)^ (AIC and BIC), which penalise for the number of parameters in a model. The knots were placed at percentile values according to Harrell ^[[3]](#footnote-3)^. Departures from linearity were assessed with a likelihood ratio test (LRT) ^[[4]](#footnote-4)^ by comparing models including and excluding the nonlinear components of the splines, with p < 0.05, indicating that the nested model with the nonlinear component had a better fit.

1. Akaike, H. (1973). Information theory and an extension of the maximum likelihood principle. In: Petrov, B.N., Csáki, F. (Eds.), 2nd International Symposium on Information Theory. Akadémia Kiadó, Budapest, pp. 267–281. [↑](#footnote-ref-1)
2. Akaike, H., (1978). A Bayesian analysis of the minimum AIC procedure. Annals of the Institute of Statistical Mathematics 30:9–14. [↑](#footnote-ref-2)
3. Harrell Jr FE. Regression modeling strategies: with applications to linear models, logistic regression, and survival analysis. New York: Springer; 2001. [↑](#footnote-ref-3)
4. Neyman J, ES P. On the use and interpretation of certain test criteria for purposes of statistical inference. Biometrika, 1928. 20A: p. 175-240, 263-97. [↑](#footnote-ref-4)
