# Supplementary material for: Association of pre-eclampsia risk with maternal levels of folate, homocysteine and vitamin B12 in Colombia: A case-control study
Source: PLoS One. 2018 Dec 6;13(12):e0208137. doi: 10.1371/journal.pone.0208137 (PMC6283543; doi:10.1371/journal.pone.0208137)
Supplement: S1 Table — (DOCX) [file pone.0208137.s002.docx]

**SUPPORTING INFORMATION**

| **S1 Table.** **Values outside the assay detection limit (DL) for homocysteine, folate and vitamin-B12 maternal levels.** | | | | | | |
| --- | --- | --- | --- | --- | --- | --- |
|  | **Cases** | **Controls** |  | **Cases** | **Controls** |  |
| **Variable** | **Below DT**  **n (%)** | **Below DT**  **n (%)** | ***p**** | **Above**  **DT**  **n (%)** | **Above**  **DT**  **n (%)** | ***p**** |
| Homocysteine (μmol/L) | 19/1103 (1.7) | 32/1291 (2.5) | 0.202 | 1/1103 (0.1) | 0/1291 (0.0) | 0.279 |
| Folate (ng/mL) | 11/1101 (1.0) | 15/1287 (1.2) | 0.696 | 22/1101 (2.0) | 16/1287 (1.2) | 0.142 |
| Vitamin B12 (pg/mL) | 376/1099 (34.2) | 499/1289 (38.7) | 0.023 | 18/1099 (1.6) | 5/1289 (0.4) | 0.002 |

*p-values were calculated by Chi-squared test by comparing the proportion of values below/above DL between cases and controls.
