# Supplementary material for: Association of pre-eclampsia risk with maternal levels of folate, homocysteine and vitamin B12 in Colombia: A case-control study
Source: PLoS One. 2018 Dec 6;13(12):e0208137. doi: 10.1371/journal.pone.0208137 (PMC6283543; doi:10.1371/journal.pone.0208137)
Supplement: S2 Table — (DOCX) [file pone.0208137.s003.docx]

**SUPPORTING INFORMATION**

| **S2 Table. Folic acid supplementation of participants from the GenPE study during pregnancy.** | | | | | | | | |
| --- | --- | --- | --- | --- | --- | --- | --- | --- |
| **First trimester** | **-** | **+** | **-** | **-** | **+** | **+** | **-** | **+** |
| **Second trimester** | **-** | **-** | **+** | **-** | **+** | **-** | **+** | **+** |
| **Third trimester** | **-** | **-** | **-** | **+** | **-** | **+** | **+** | **+** |
| Cases (n) | 345 | 356 | 611 | 154 | 275 | 17 | 539 | 356 |
| Controls (n) | 467 | 393 | 587 | 235 | 360 | 11 | 803 | 552 |
| **Supplementation group** | **Reference** | **1 to 2 trimesters** | | | | | | **All pregnancy period** |

A negative sign (-) corresponds to women reporting not having consumed folic acid during that trimester. A positive sign (+) corresponds to women reporting having consumed folic acid during that specific trimester
