# Supplementary material for: Association of pre-eclampsia risk with maternal levels of folate, homocysteine and vitamin B12 in Colombia: A case-control study
Source: PLoS One. 2018 Dec 6;13(12):e0208137. doi: 10.1371/journal.pone.0208137 (PMC6283543; doi:10.1371/journal.pone.0208137)
Supplement: S3 Table — (DOCX) [file pone.0208137.s004.docx]

**SUPPORTING INFORMATION**

| **S3 Table. Comparison between models for the association of maternal vitamin B12 with pre-eclampsia using imputed and not-imputed data.** | | | | | | |
| --- | --- | --- | --- | --- | --- | --- |
| Vitamin B12 data | n | Unadjusted  OR (95% CI) | n | Minimal  adjustment  OR (95% CI) | n | Full  adjustment  OR (95% CI) |
| Excluding values below DL | 1501 | 1.49  (1.25, 1.78) | 1500 | 1.27  (1.04, 1.55) | 1437 | 1.25  (1.02, 1.53) |
| Simple imputation (DL value) | 2374 | 1.25  (1.15, 1.36) | 2373 | 1.11  (1.01, 1.22) | 2270 | 1.11  (1.01, 1.23) |
| Simple imputation (1/2 DL value) | 2374 | 1.20  (1.11, 1.31) | 2373 | 1.06  (0.97, 1.17) | 2270 | 1.07  (0.97, 1.18) |
| Multiple imputation | 2374 | 1.23  (1.13, 1.34) | 2373 | 1.08  (0.97, 1.19) | 2298 | 1.10  (0.99, 1.22) |

Minimal adjustment: maternal age, gestational age, recruitment centre, and ethnicity.

Full adjustment: minimal adjustment plus recruitment date in year-over-year increments, multiple pregnancy, smoking, socioeconomic position, and infections during pregnancy.

DL: detection limit.
