# Supplementary material for: Association of pre-eclampsia risk with maternal levels of folate, homocysteine and vitamin B12 in Colombia: A case-control study
Source: PLoS One. 2018 Dec 6;13(12):e0208137. doi: 10.1371/journal.pone.0208137 (PMC6283543; doi:10.1371/journal.pone.0208137)
Supplement: S1 Fig — (DOCX) [file pone.0208137.s005.docx]

**SUPPORTING INFORMATION**

**S1 Fig.** **Path diagram association (Directed Acyclic Graph - DAG) between folic acid supplementation and maternal folate levels with pre-eclampsia**. This diagram shows a direct pathway of the effect of folic acid supplementation on the development of pre-eclampsia and an indirect pathway, in which the effect of folic acid supplementation on pre-eclampsia is mediated by the concentration of acid folic in blood. Pink arrows indicate biasing path and green arrows causal path.


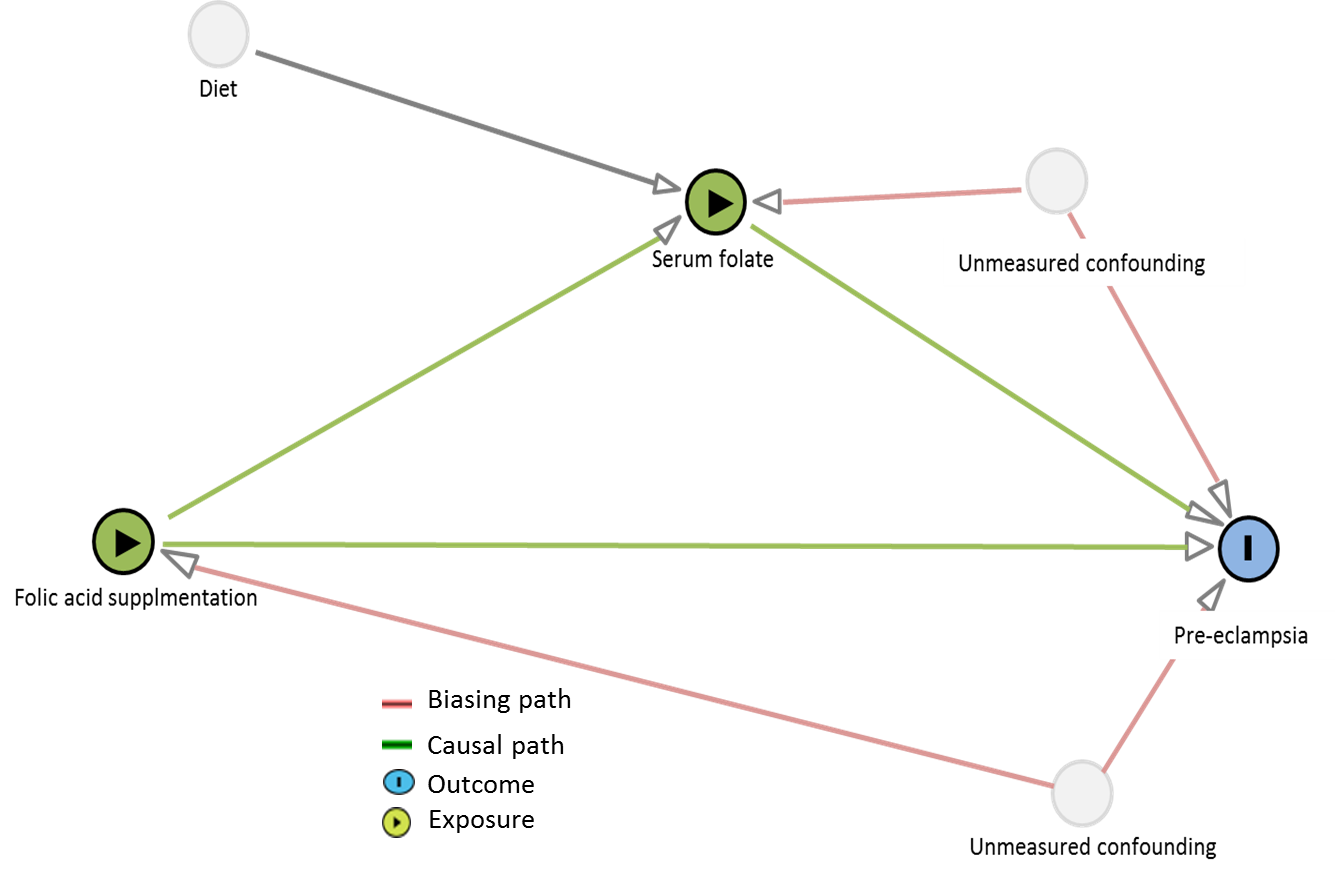


*Source: Own elaboration through the website: http://www.dagitty.net/development/dags.html*
